# Comparison of three next-generation sequencing platforms for metagenomic sequencing and identification of pathogens in blood

**DOI:** 10.1186/1471-2164-15-96

**Published:** 2014-02-04

**Authors:** Kenneth G Frey, Jesus Enrique Herrera-Galeano, Cassie L Redden, Truong V Luu, Stephanie L Servetas, Alfred J Mateczun, Vishwesh P Mokashi, Kimberly A Bishop-Lilly

**Affiliations:** 1 Naval Medical Research Center, NMRC-Frederick, 8400 Research Plaza, Fort Detrick, Frederick, MD 21702, USA; 2 Henry M. Jackson Foundation, 6720-A Rockledge Drive, Suite 100, Bethesda, MD 20817, USA; 3 Department of Microbiology and Immunology, Uniformed Services University, Bethesda, MD 20814, USA

## Abstract

**Background:**

The introduction of benchtop sequencers has made adoption of whole genome sequencing possible for a broader community of researchers than ever before. Concurrently, metagenomic sequencing (MGS) is rapidly emerging as a tool for interrogating complex samples that defy conventional analyses. In addition, next-generation sequencers are increasingly being used in clinical or related settings, for instance to track outbreaks. However, information regarding the analytical sensitivity or limit of detection (LoD) of benchtop sequencers is currently lacking. Furthermore, the specificity of sequence information at or near the LoD is unknown.

**Results:**

In the present study, we assess the ability of three next-generation sequencing platforms to identify a pathogen (viral or bacterial) present in low titers in a clinically relevant sample (blood). Our results indicate that the Roche-454 Titanium platform is capable of detecting Dengue virus at titers as low as 1X10^2.5^ pfu/mL, corresponding to an estimated 5.4X10^4^ genome copies/ml maximum. The increased throughput of the benchtop sequencers, the Ion Torrent PGM and Illumina MiSeq platforms, enabled detection of viral genomes at concentrations as low as 1X10^4^ genome copies/mL. Platform-specific biases were evident in sequence read distributions as well as viral genome coverage. For bacterial samples, only the MiSeq platform was able to provide sequencing reads that could be unambiguously classified as originating from *Bacillus anthracis.*

**Conclusion:**

The analytical sensitivity of all three platforms approaches that of standard qPCR assays. Although all platforms were able to detect pathogens at the levels tested, there were several noteworthy differences. The Roche-454 Titanium platform produced consistently longer reads, even when compared with the latest chemistry updates for the PGM platform. The MiSeq platform produced consistently greater depth and breadth of coverage, while the Ion Torrent was unequaled for speed of sequencing. None of the platforms were able to verify a single nucleotide polymorphism responsible for antiviral resistance in an Influenza A strain isolated from the 2009 H1N1 pandemic. Overall, the benchtop platforms perform well for identification of pathogens from a representative clinical sample. However, unlike identification, *characterization* of pathogens is likely to require higher titers, multiple libraries and/or multiple sequencing runs.

## Background

Metagenomic sequencing involves high-throughput sequencing of complex samples comprised of nucleic acid from multiple organisms. Although the technique is relatively new, published examples of metagenomic sequencing to detect and/or characterize a causal agent in diseases of animals and humans are already too numerous to be summarized here. In some cases, as in the case of a novel filovirus that caused an outbreak in Uganda in 2007, metagenomic sequencing was used to follow up when results from traditional diagnostic assays indicated a novel agent. For instance, the Ugandan filovirus had produced a positive result in a broadly cross-reactive IgM capture assay followed by mixed results in RT-PCR assays for known filoviruses, and so metagenomic sequencing was employed to characterize the genome of the novel virus [1]. Another example whereby more traditional diagnostic assays and metagenomic sequencing both played a role in detection is the identification of human metapneumovirus causing fatal infection of wild Rwandan gorillas [2]. The opposing scenario, in which traditional diagnostic assays completely fail and metagenomic sequencing plays more than just a supporting role, includes a recent report of astrovirus encephalitis in an immune-compromised teenage boy [3].

Metagenomic sequencing has many advantages over more traditional methods of pathogen detection, such as PCR, ELISA, cytopathic effect, etc. These advantages include relative speed, the ability to detect non-culturable organisms, and, perhaps most importantly, the fact that metagenomic sequencing requires little to no *a priori* knowledge of the organism(s). However, despite these advantages, there are some significant challenges involved. These difficulties may include computationally intensive analyses; in some cases the lack of appropriate reference genomes for comparison; difficulties in sample preparation, including matrix effects and biases introduced from extraction; as well as the need for sufficient depth and breadth of coverage to detect pathogens at potentially very low levels in a given sample. It is also important to note that there are currently no standards in terms of what constitutes “identification” of a pathogen in a sample. A number of organism-specific reads will be necessary to make a true positive call, but how many reads or signatures is necessary to invoke confidence is not standardized and may vary as per sample type/complexity or may be organism-dependent. It is likely that increased depth of coverage would increase confidence in a species or strain call, but again, there is no agreed upon standard regarding depth of coverage for metagenome samples, and in many cases it would be reasonable to expect that a region of a pathogen genome detected within a metagenome sample may be only present within the reads at 1X coverage.

Just as there is currently no agreed upon standard to indicate what breadth or depth of coverage would be required to make an “identification,” there is currently a paucity of knowledge regarding the actual limits of detection (LoD) for each sequencing platform and protocol. Detection of even one or several pathogen-specific reads in a clinical sample that are not found in control samples is likely to be interpreted as a positive result. However, in the absence of LoD data, it is difficult to conclude with any confidence that a pathogen is not present simply because no pathogen-specific reads are detected. Recently, Moore et al. conducted a LoD study whereby viral RNA in serial dilutions was spiked into a colorectal biopsy sample and sequenced using the Illumina platform. In this study, although the proportion of viral reads detected was less than expected, potentially due to issues quantitating the RNA, virus-specific reads were detected from samples spiked with less than picogram quantities [4]. In another study, Cheval et al. spiked plasma and cerebrospinal fluid with known concentrations of eleven different viruses to assess the level of detection by Roche-454 pyrosequencing as compared to Illumina, and found that the higher output (number of reads) produced by the Illumina platform resulted in better detection of the viruses per run. The authors report detection of viruses by their 454 sequencing at titers of 1X10^3^*pfu/ml* and higher, and by Illumina of viral genomes present at 1X10^2.4^*genome copies/ml* and higher [5].

Other studies have suggested that the LoD by 454 sequencing for an RNA virus with a genome size of ~10-11 kb lies between 1X10^2^ and 1X10^3^ pfu/mL [6]. Likewise, in a recent study using artificially constructed marine metagenomes, Pochon et al. determined that the 454 GS Junior was capable of detecting DNA from species present at levels greater than 0.64% of the whole. It should be noted, however, that this study used amplicons from a single gene to determine sensitivity [7]. Finally, variation in extraction techniques and/or sample preparation kits have been shown to affect the composition of sequence reads [8,9]. Thus, any reported LoD is likely to be very protocol specific, as modifications introduced at each step of the procedure can have drastic effects on overall sequence read output and quality.

In a previous study, we assessed the ability of the Roche-454 platform to detect low-level pathogens present in a complex host background, insect vectors [6]. Results indicated that pathogen reads present in proportions of 0.1% were sufficient to cover 90% of a viral genome at a depth of >4X. The same study postulated the number of sequence reads required to identify a pathogen present in a sample in which 1 out of 100 pooled mosquitoes is infected. The projected number of reads necessary was expected to vary depending on titer, with high titer infections requiring hundreds of thousands of reads, while low titer infections would require tens of millions. In the current study we use the latest sequencing technologies with their increased throughput and lower cost to confirm these projections as well as establish metrics for pathogen identification from clinical samples.

## Results

### Limits of detection of Roche-454 pyrosequencing

Our previous study suggested that as few as 0.1% of the total reads were sufficient to unambiguously identify a viral pathogen present in a complex background [6]. However, our experimental model was reflective of a disseminated infection and high viral load [6,10-12]. Therefore, we wished to determine if the Roche-454 platform was capable of detecting viruses at reduced titers.

**Figure 1:**
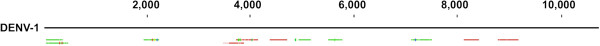
**Read mapping against DENV-1 genome at 1X10**^**2.5 **^**pfu/ml.** Reads resulting from 1 454 Titanium sequencing run of a cDNA library made from blood spiked with DENV-1 at titer of 1X10^2.5^ pfu/ml were aligned to the reference genome NC_001477.1 using CLC Genomics Workbench version 6.0.4 at default parameters.

To determine the limits of detection (LoD), mock samples were created by spiking known amounts of Dengue virus Type 1 (DENV-1) into 1 mL aliquots of whole human blood. Total RNA was extracted from each sample and used for construction of cDNA libraries. Sequencing of samples representing each titer was conducted in replicate fashion on the Roche-454 platform and DENV-1 was detected in a range of titers from 1X10^3^ to 1X10^5^ pfu/ml. Since no DENV-1 specific reads were detected at the titer of 1X10^2^, it was determined that the effective LoD for 454 sequencing using this protocol must lie between 1X10^2^ and 1X10^3^, and so several samples were prepared within this range (1X10^2.3^, 1X10^2.5^, and 1X10^2.7^). Sequencing was initiated with the 1X10^2.5^ sample and progressed down to 1X10^2.3^, from which no reads were detected. Overall, the decrease in the number of viral reads approximated the reduction in titer, especially at lower titers. At the lowest titer detectable, 1X10^2.5^, the breadth of coverage of the reference genome was 31% with an average depth of coverage of 0.35X upon read mapping (Figure 1). In a parallel approach, *de novo* assembly of reads yielded a single contig of 427 bp in length. By comparison, assemblies of samples at 1X10^5^ pfu/mL and 1X10^4^ pfu/mL yielded one contig of 9445 bp and two contigs of 4640 and 3566 bp, respectively. In both cases the largest contig in the *de novo* assembly belonged to DENV-1. It is striking that, in the case of the low titer samples, *de novo* assembly of the reads provides less information than would a taxonomic classification of all reads. In the case of the 1X10^2.5^ pfu/mL sample, although 11/604,130 resulting reads map to the reference, covering 31% of the viral genome, *de novo* assembly of the reads produces a single contig comprised of 2 reads that covers only 4% of the viral genome (Table 1). This apparent difference between fraction of the viral genome covered by the *de novo* assembly and the reference mapping is due to insufficient breadth of coverage for the reads to overlap and be used in *de novo* assembly.

**Table 1:** Roche-454 read statistics for DENV-1 at multiple titrations

**Dengue-1 pfu/mL**	**Estimated max. genome copies/mL**	**Reads mapped per 100,000**	**Fraction of reference covered**	**Average C**_**t**_	**Contig(s) by *de novo *** **assembly?**	**Fraction of reference covered**
1X10^2^	1X10^4^ – 1.7X10^4^	0	0	NA	N	0
1X10^2.3^	2X10^4^ – 3.4X10^4^	0	0	27.65	N	0
1X10^2.5^	3.2X10^4^ – 5.4X10^4^	0.34	0.05	ND	N	0
1X10^2.5^	3.2X10^4^ – 5.4X10^4^	1.82	0.31	27.03	Y	0.04
1X10^3^	1X10^5^ – 1.7X10^5^	3.4	0.4	25.66	Y	0.20
1X10^3^	1X10^5^ – 1.7X10^5^	4.7	0.72	ND	ND	ND
1X10^4^	1X10^6^ – 1.7X10^6^	34.2	0.92	21.79	Y	0.76
1X10^5^	1X10^7^ – 1.7X10^7^	190.9	0.99	18.05	Y	0.99
1X10^6^	1X10^8^ – 1.7X10^8^	227.1	0.99	14.56	Y	0.99

### Comparison of Roche-454, Illumina MiSeq and Ion Torrent PGM for detecting viral pathogens

Next, we examined how the performance of benchtop sequencers would compare with that of the Roche-454. To that end, mock samples containing Influenza virus H1N1 were prepared in a similar fashion to the DENV-1 samples. For this experiment, the DENV-1 results were used as a guide as to what range of titers to investigate. The effective LoD for the 454 platform was found to be 1X10^2.5^, which we estimate to correlate with an upper limit of 31,600-53,720 genome copies/ml, based on work by Houng et al. and Wang et al., who found one DENV pfu to correspond to 100 and 170 genome copies by qPCR, respectively [13,14]. Therefore, an initial titer of 1X10^4^ - genome copies/mL was chosen for these experiments. At 1X10^4^ genome copies/mL the Roche-454 platform was capable of detecting the Influenza virus in only one out of two replicates, with only a single hit covering 3% of the flu genome (Table 2). At the same concentration, two biological replicates sequenced on the PGM platform scored 11 and 21 hits, covering 7% and 12% of the genome, respectively. By contrast, two biological replicates sequenced on the MiSeq platform scored 308 and 811 hits, covering 79% and 95% of the genome, respectively. Interestingly, even when the number of Influenza-specific reads is expressed as reads per 100,000, the MiSeq and PGM platforms both outperform the Roche-454, indicating that there may be some platform-specific factors that affect LoD in addition to total output per run.

**Table 2:** Cross-platform comparison of read mapping to Influenza A H1N1

**Platform**	**#/Replicates**	**Reads mapped (low)**	**Reads mapped per 100,000**^**a**^	**Reads mapped (med)**	**Reads mapped in pairs**^**a**^	**Fraction of reference covered**^**a**^
PGM 1	3	11/20/ND	.61/.80/ND	5/16/ND	NA	.07/.15/ND
PGM 2	3	21/19/18	.87/1.3/.86	14/11/10	NA	.12/.09/.14
Roche	2	1/0	.17/0	0/0	NA	.03/-
MiSeq 1	3	308/322/323	.82/.90/1.0	269/311/318	66/72/82	.79/.95/.80
MiSeq 2	3	811/942/912	2.2/2.4/2.2	741/934/914	296/216/248	.95/.95/.97

a: Reads mapped at low stringency.

**Figure 2:**
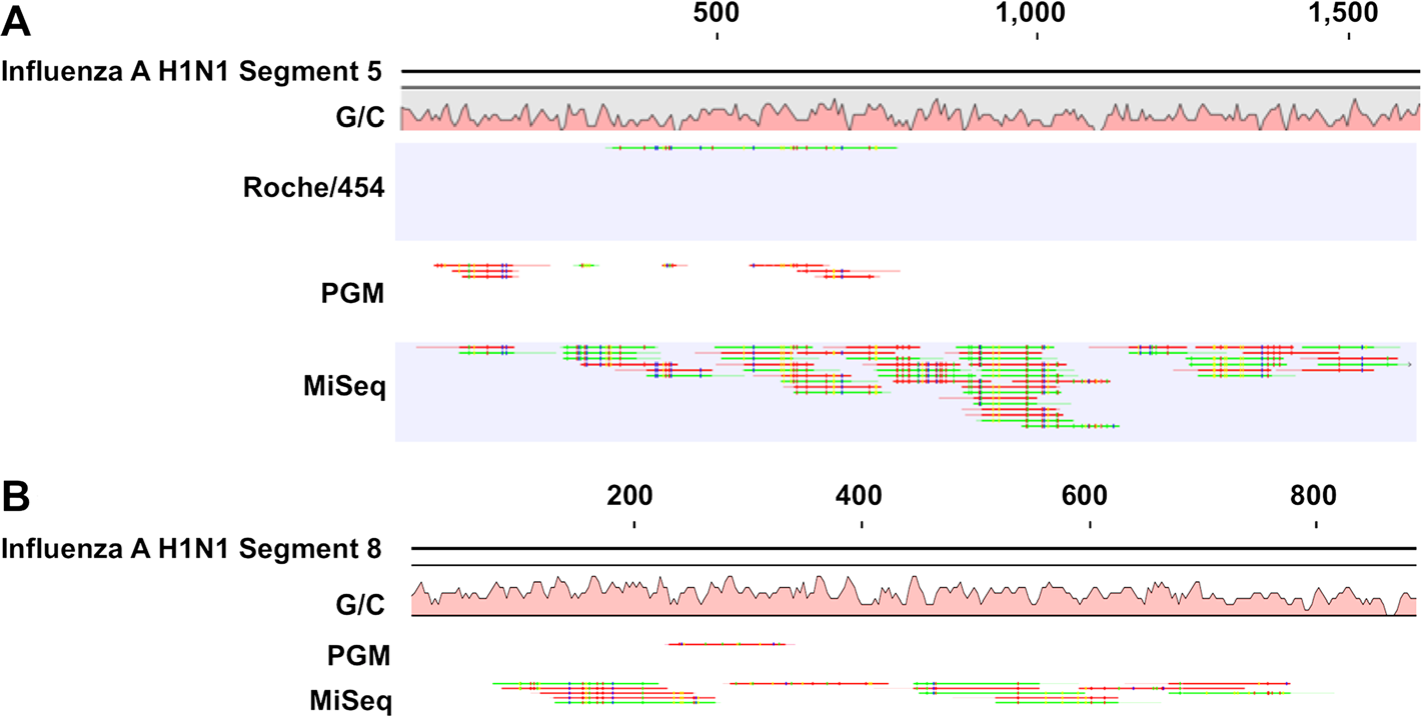
**Read mapping against representative segments of Influenza genome.** In **A)**, reads resulting from a representative run of 454 Titanium sequencing, Ion Torrent PGM sequencing, and Illumina MiSeq sequencing were mapped to the reference Influenza A H1NI segment 5 [NC_002019.1], using CLC Genomics Workbench version 6.0.4 at default parameters, whereas in **B)**, they were mapped against segment 8 [NC_002020.1]. In **B)**, no Roche-454 reads mapped to the reference. In both **A)** and **B)**, coordinates of reference genome segment are displayed along the top and G/C content is graphed below reference in pink.

Examination of the read mappings revealed that the mapped reads were not evenly distributed along the viral genome for any of the three sequencing platforms. Figure 2A depicts representative read mappings by platform for segment 5 of Influenza A H1N1. For the PGM, the regions of highest coverage were found most often toward the 5′ end of segment 5. Specifically, 14/19 mapped reads aligned within the first quarter of the genome segment. The single mapped read from the Roche-454 was 448 bases in length. By contrast, the longest mapped read for the PGM was 179 bases. As expected, the mapped reads from the MiSeq platform were all 150 bases in length. Since the PGM offers scalability in throughput, we sought to compare the sensitivity of the PGM using a 314 chip as well. The throughput of the 314 chip is similar to that of one-half of a 454 picotitre plate. Although not shown in Table 2, two independent experiments using a 314 chip yielded a single, 84 base read mapped to Segment 5 (Additional file 1: Figure S1). This single read mapped within the first quarter of Segment 5. These data strongly suggest that increased throughput is required for detection of pathogens present at low levels.

A closer examination of the read mappings for both the PGM and MiSeq platforms indicated that some mapped reads were spurious. In order to eliminate these matches, stringency of read mapping parameters was increased. At the medium stringency setting, whereby the length of the read required to match was increased from 50% to 70%, the number of mapped reads dropped by roughly half for the PGM and by roughly 10% for the MiSeq. Furthermore, the number of MiSeq reads that mapped in pairs was roughly 25% (66 out of 269; Table 2). This is most likely due to polymorphisms in the sequenced strain that are not present in the NCBI reference genome. As expected, no reads mapped at the highest level of stringency tested, as the requirement for 100% identity would preclude any matches.

For Influenza virus segment 8, the MiSeq yielded 14 mapped reads versus the single mapped read from the PGM. It is noteworthy that although the MiSeq reads covered more of the segment overall than the single PGM read did, both platforms produced a single read in an area of high G/C content. Given that the MiSeq consistently produced the highest depth of coverage, we next examined whether the increased coverage of the MiSeq was sufficient to determine the presence of a single nucleotide polymorphism (SNP) that conveys resistance to a widely used antiviral drug, oseltamavir (Ose). Previous studies have established that Influenza A strains from the 2009 epidemic had mutated to an Ose-resistant phenotype [15]. Thus, we examined the reads resulting from MiSeq sequencing of a 2009 epidemic strain for the presence of the mutation H275Y, and in neither of two biological replicates were any of the known SNPs detected. In fact, there was zero coverage of that specific region of the genome. Given that our experimental model, human blood, represents a relatively low-complexity sample (as compared to clinical samples from other body sites such as oropharyngeal swabs or fecal samples), spiked with Influenza A genomes at titers higher than would be expected to be encountered in a clinical sample, these data suggest that pathogen enrichment may be a necessary step for determination of drug resistance by metagenome sequencing.

### *de novo* assembly and BLASTn analysis of Influenza A reads

In many cases the identity of a pathogen may not be known or the pathogen’s genome may be very divergent at the nucleic acid level from that of known family members, necessitating analysis by means other than mapping reads to a reference genome. Therefore, *de novo* assemblies of the Influenza A metagenomes from the PGM and MiSeq data were performed, followed by BLASTn analysis. Assembly of the PGM reads using MIRA produced an average of 28,000 contigs, yet not a single contig was classified as Influenza A (Table 3). In fact, no contig was classified as a viral taxon. This result is surprising given the observed sequence depth in several regions of the Influenza A genome (see Figure 2A). We also attempted assembly of the PGM reads using Velvet with similar assembly parameters. Again, none of the resultant contigs were able to be assigned to Influenza A. Upon closer inspection, it was observed that the identified reads were either substrings of each other and therefore absorbed during the merge step of assembly, or they had a stretch of mismatches long enough (≥ 10 bases) to cause the assembly algorithm to mark these reads as not overlapping.

**Table 3:** ***De novo *assembly and BLASTn statistics of Influenza A sequence reads**

**Platform**	**Assembler**	**# total contigs**	**# specific contigs**
PGM 1	MIRA	26,171	0
PGM 2	MIRA	30,719	0
MiSeq 1	Velvet	841,384	18
MiSeq 2	Velvet	768,912	11

By comparison, assembly of the MiSeq reads for two biological replicates using Velvet generated well over 750,000 contigs for both replicates, with 18 and 11 flu-specific contigs, respectively. Taxonomic classification of the contigs by MEGAN properly assigned the correct strain (Influenza A virus (A/swine/Iowa/15/1930(H1N1)) in 5 out of 18 and 0 out of 11 contigs. Importantly, only the strain level classifications were mis-assigned. In each case, the correct genus and species calls were made. Therefore, each of the contigs identified by MEGAN as Influenza but not correctly identified at strain level was individually subjected to BLASTn analysis and manual curation of results. This inspection revealed that the proper strain was indeed present in the hit tables (6 and 7 contigs, respectively), but that for each of these contigs, there were several strains hit with the identical E-value, including A/swine/Iowa/15/1930(H1N1), and therefore, a definitive strain call could not be made in those cases. Thus, for one of two replicates, a majority of the influenza-specific contigs resulting from *de novo* assembly of the MiSeq reads were either properly classified to the strain level, or conserved amongst a subset of strains including A/swine/Iowa/15/1930(H1N1). It was interesting to note that, in each case, contigs corresponding to Segments 4 (HA) and 6 (NA) were not assigned to the proper strain. These results demonstrate that the increased throughput of the MiSeq enables species level identification of reads present in proportions as low as .0008% of the total.

### Comparison of Influenza A sequencing replicates on the PGM and MiSeq

We were interested in further examining the observed differences in the number of mapped reads between biological replicates and between genome segments. In addition, we wished to determine the reproducibility of our results. The biological replicates (derived from independent spikings and library preparations) sequenced on the MiSeq platform exhibited considerable variability (Table 2), and we were interested in determining at what stage this variability would arise- at library construction or sequencing. Thus, we performed replicate sequencing runs for each of the PGM and MiSeq libraries. The total number of mapped reads for each technical replicate was quite consistent (Table 2). This observation held true for both platforms. Moreover, each individual library yielded a number of mapped reads that was less than one standard deviation from the average. Thus, the inter-run variability seems to be limited and results of sequencing a given library preparation are highly reproducible.

However, we did note several tendencies regarding the number of mapped reads per segment (Additional file 2: Table S1 and Additional file 3: Table S2). Firstly, segment 5 tended to be overrepresented in each PGM replicate. Specifically, for comparison, we expressed the proportion of mapped reads for a given genome segment as a function of that particular segment’s length, as follows:

$$\begin{array}{l} y=\left(\#\text{mapped}\;\text{reads}\;\text{per}\;\text{given}\;\text{segment}/\#\text{total}\;\text{reads}\;\text{mapped}\right)\\ \;/\text{segment}\;\text{length}\times 1000 \end{array}$$

**Figure 3:**
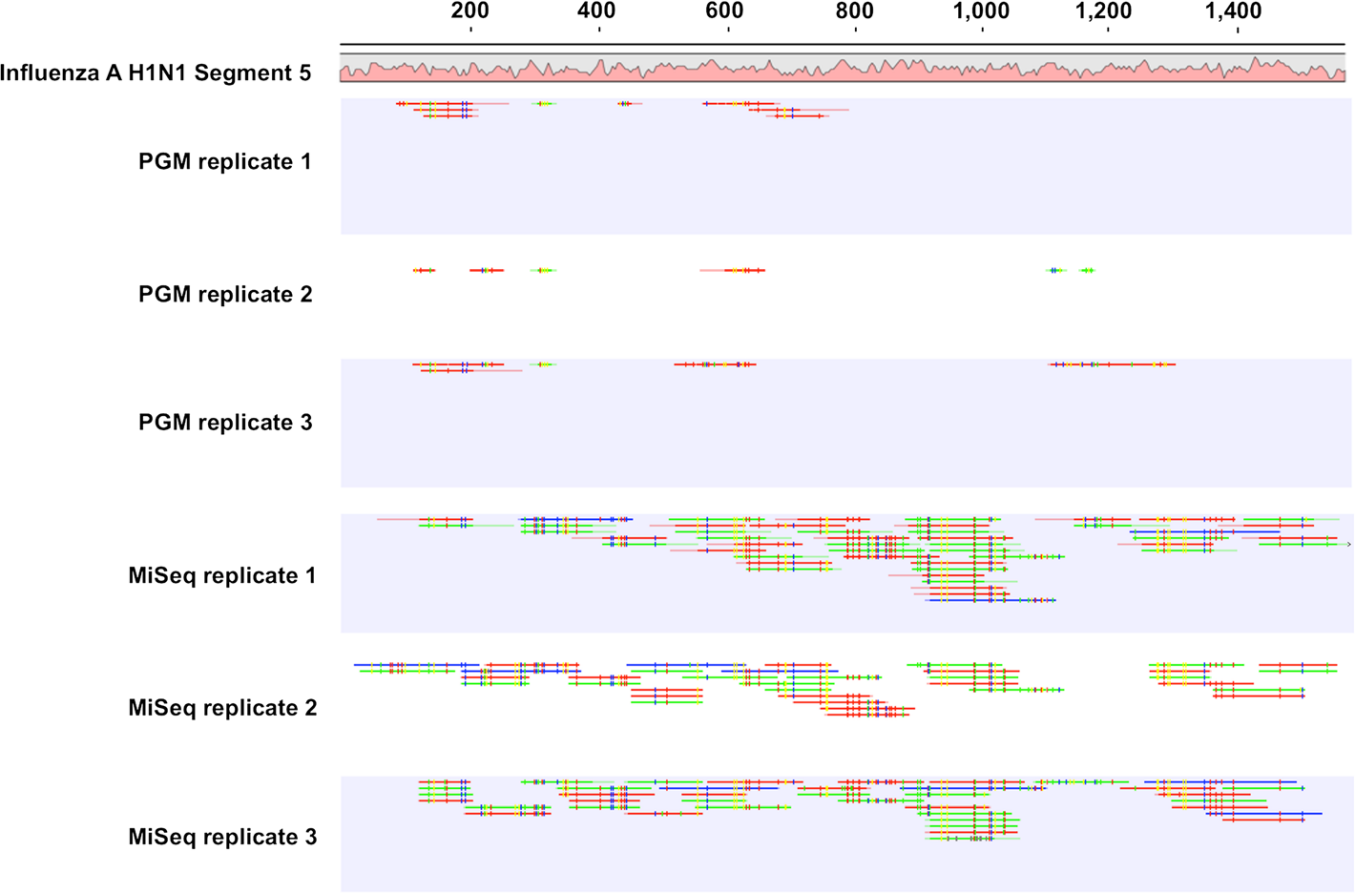
**Read mapping against segment 5 of Influenza A of replicate sequencing runs.** Reads resulting from a replicate run of Ion Torrent PGM sequencing (top) and Illumina MiSeq sequencing (bottom) were mapped to the reference Influenza a H1NI segment 5, [NCBI accession: NC_002019 ] using CLC Genomics Workbench version 6.0 at default parameters. Coordinates of reference genome segment are displayed along the top and G/C content is graphed below reference in pink.

This value for segment 5 is more than twice that for any other segment by PGM sequencing (Additional file 3: Table S2). This is an interesting observation given that segment 5, at 1565 bases in length, is the fifth largest segment in the genome. In every case for the PGM, segment 5 yielded the most mapped reads. This observation is consistent with our previous result with the 314 chip in which the only segment mapped was segment 5 (Additional file 1: Figure S1). Secondly, segment 3 tended to be underrepresented, in both the PGM and MiSeq data. This trend was especially marked in the PGM libraries as compared to the MiSeq libraries (.03 and .05 respectively). These findings suggest that some sequences are inherently favored for sequencing by the PGM as well as the MiSeq. Thirdly, regardless of the number of reads mapped, the coordinates of the mapped reads in relation to the genome were widely variable (Figure 3). This effect was more pronounced in the PGM libraries. This suggests that the library diversity is greater than that sampled by a single sequencing run.

### Comparison of Roche-454, Illumina MiSeq and Ion Torrent PGM for detecting bacterial pathogens

Although there have been performance comparisons of the benchtop platforms for sequencing bacterial isolates [16,17] to our knowledge, there has been no published report of comparisons of sensitivity and specificity in terms of speciation. Therefore, we aimed to establish a LoD for each sequencer using samples spiked with a known biothreat agent, *Bacillus anthracis.* Using the Sterne strain as a surrogate of anthrax infection, the quantity of bacterial cells present in a dilution series was established. Using three complementary quantitative techniques, mock samples with *B. anthracis* Sterne present at roughly 30,000 cells/mL of human blood were prepared.

All three platforms delivered sequence reads matching the chromosome of *B. anthracis* Sterne by reference mapping (Table 4). As expected, given the increased throughput, both PGM and MiSeq yielded significantly higher numbers of reads as well as genome coverage. However, at low stringency, large numbers of reads were falsely counted as mapped. This trend was especially prominent in the PGM data. Most of the spurious reads were short (average: 40 bases) and low-complexity (i.e. rich in nucleotide repeats). This tendency was confirmed in the replicate samples. The same pattern was observed in the Roche-454 sequence reads as well. In general, the 454 reads were clustered near the 5′-end of the linear reference sequence, while the PGM and MiSeq reads spanned a much larger breadth. However, the 454 reads were uniformly longer and more information-rich. The MiSeq replicates demonstrated a similar tendency as that of the PGM; in general, the lower stringency mapping contained matched reads of short length (avg. 66 bases). For comparison, in replicate two for both platforms, 80.77% of mapped MiSeq reads mapped as non-perfect matches versus 98.83% of mapped PGM reads. The average read length of a non-perfectly matched MiSeq read was 53.22 bases, as compared to 39.99 bases for the PGM. Interestingly, the mean number of mismatches per read tended to be higher in the MiSeq data than that of the PGM (mean of 26 for MiSeq vs. 15 mismatches for PGM). Due to the slightly longer length of mapped MiSeq reads, there could conceivably be more opportunity for mismatched bases per read. Therefore, we compared the mismatches within MiSeq reads and PGM reads by expressing mean number of mismatches per read as a proportion of mean read length of non-perfectly matched reads. When expressed in this manner, the proportion of mismatches per length of non-perfectly matched reads is 0.5 for the MiSeq versus 0.4 for the PGM. Additionally, the number of indel related mismatches was found to be proportionally higher in the MiSeq reads than in the PGM reads. Conversely, the PGM reads had a higher number of A → T and G → C transversions than the MiSeq data. This is inconsistent with the known error models for both of these platforms.

**Table 4:** **Cross-platform Comparison of Read Mapping to *B. anthracis *Sterne**

**Platform**	**#Replicates**	**Reads mapped (low)**	**Reads mapped (med)**	**Reads mapped (high)**	**Fraction of reference covered**^**a**^	**Detected by qPCR?**
PGM	2	29,534/15,676	7,689/4,286	247/178	.12/.09	Y
Roche-454	2	384/376	249/240	65/56	.01/.01	Y
MiSeq	2	10,415/41,242	3,024/9,985	1,633/7,930	.07/.19	Y

a: Fraction of reference covered using high stringency mapping parameters as defined in Methods.

Although *de novo* assemblies and taxonomic classification were attempted for both PGM and 454 reads, no dataset produced a single contig that matched *Bacillus spp.* This is most likely due to the lack of coverage of the bacterial genome, resulting in a lack of sufficient overlap between reads necessary to form a contig. By comparison, *de novo* assembly of one MiSeq run yielded 6,140,965 total contigs. Taxonomic classification of the assembled sequence reads rendered 1200 contigs that were assigned to the *Bacillus cereus* superfamily. More importantly, 70 contigs were properly classified as *Bacillus anthracis* Sterne. These results echo those of the viral samples in that assembled sequence reads from the MiSeq were able to identify the spiked-in microorganism, in this case to the strain level.

### Detection of genetic engineering

**Figure 4:**
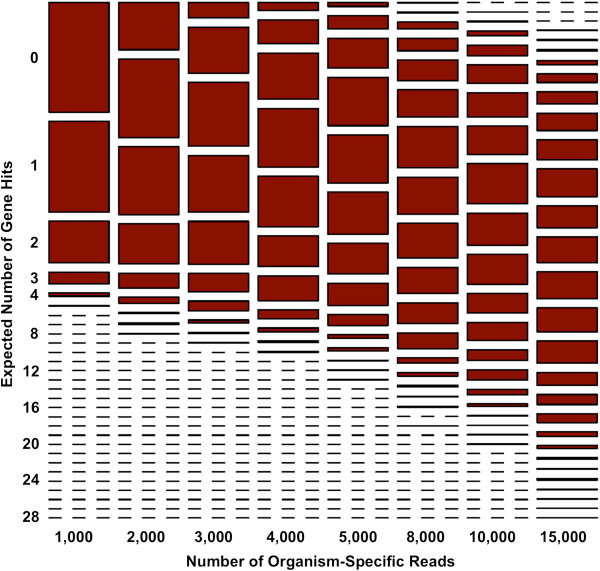
**Mathematical modeling of the likelihood of detecting a genetic modification in *B. anthracis.*** The expected number of hits to an inserted gene of size 1 kb, at 5 copies, was simulated as a function of the number of organism-specific reads collected from the metagenomic sample. The relative size of each rectangle indicates the proportion of samplings for which a specific number of hits is expected.

We were interested in whether sequencing reads near the LoD could detect an instance of genetic engineering. Therefore, the strain of *B. anthracis* spiked into human blood for these experiments was a 34F2 strain of *B. anthracis* containing a stably integrated genomic copy of red fluorescent protein (RFP). Metagenomic sequence data was compared with a computer simulation of the likelihood of detecting this genetic manipulation. For this purpose, we developed a Perl-based algorithm to generate a distribution of the number of sequence reads necessary to detect a gene of 1000 bps inserted at five locations into a prokaryotic genome of 5 Mb in size. At 1000 organism-specific reads, the probability of detecting the inserted gene is roughly one-third (Figure 4). In fact, the minimum number of organism-specific hits necessary to ensure detection of the inserted gene is 10,000. Given that 5 of the 6 metagenomes sequenced here contained far fewer than 1000 bacillus-specific reads (Table 4), one would expect few, if any, sequence reads to match the RFP reference sequence. Indeed, read mapping to the available NCBI reference (EF606900.1) yielded zero matches to the RFP gene present in the 34F2 chromosome. These data suggest that increased titres, targeted enrichment, or both will be necessary for detection of gene-insertion events.

## Discussion

Historically, identification of causal agents of disease has relied heavily on one’s ability to culture the organism in the laboratory and/or the use of organism-specific antibodies or sequence-based probes. However, these methods can be very limiting. For instance, some microorganisms are refractory to laboratory culture. In some cases, even microbiological assays for diseases that are manifested by cultivable organisms, such as endocarditis caused by staphylococci and streptococci, have a high false negative rate [18]. Serological assays are typically limited to identifying known or closely related organisms and antigenic drift and shift can result in false negatives. Even highly sensitive PCR-based assays must be continually updated due to signature degradation [19]. Additionally, for divergent viruses such as HIV-1, many PCR assays are unable to discriminate between closely related strains. This necessitates design of multiple probe sets, which is often a laborious task [20]. Additionally, the sensitivity of these assays is often low due to high numbers of false negatives [21]. Thus, there is a need for assays that are more robust and less pathogen specific.

Prior to the widespread adoption of high-throughput sequencing (HTS), high-density oligonucleotide microarrays were used to determine the presence of microorganisms. Syndrome-specific panels showed success in diagnosis of infectious disease [22]. However, sequence features sufficiently different from the array probe will not hybridize, resulting in false negatives. By comparison, HTS represents a relatively unbiased approach to detection of causal agents of infectious disease. However, for metagenomic sequencing to be utilized in a routine clinical context would require some basic questions answered in terms of sensitivity and reproducibility. In this study, we compared three HTS platforms for their ability to detect pathogens in human blood. As compared to the traditional Roche-454 sequencer, the benchtop sequencers IonTorrent PGM and MiSeq were better able to detect a pathogen in human blood, in part by virtue of their increased throughput.

Our reported LoD for viral samples on the Roche-454 of 1X10^2.5^ pfu/mL is similar to but slightly lower than a previously reported value of 1X10^3^ pfu/mL [5]. This may be a reflection of an increased number of reads in our study, differences in sequence library preparation or improvements in sequencing chemistry. Notably, this value is near the LoD for a validated qPCR assay [23]. Our reported LoD of 1X10^2.5^ pfu/mL corresponds to an upper limit of 31,600 – 53,720 genome copies/mL. Indeed, at that titer, the sequence reads were of sufficient number and length to unequivocally discriminate between Dengue virus subtypes. However, a strain-level designation was not possible. The inability to make a strain-level call could conceivably have potential clinical consequences. For instance, a recent phylogenetic study of circulating strains of Dengue Virus 2 indicated that a single substitution on the prM_39_ was responsible for fatal cases of Dengue Hemorrhagic Fever [24].

Using a different virus at a comparable titer, the Roche-454 platform was able to definitively identify the pathogen in only one of two replicates. Both the PGM and MiSeq surpassed the Roche-454 in terms of number of mapped reads as well as reproducibility. However, although the Roche-454 sequence data produced only a single hit, the length and quality of the hit were adequate to make a correct strain call (data not shown).

The apparent difference in the ability of each platform to detect a given pathogen is a function of the total output per platform. When the values are expressed in a normalized manner (pathogen reads per 100,000), it becomes apparent that the analytical sensitivity of the two benchtop platforms is roughly the same and surpasses that of Roche-454 (Table 2). Neither technology seems to have a significant advantage regarding detection or characterization of a pathogen if the number of reads is held constant. When cost per sequencing run or per megabase is factored in, the benchtop sequencers are a more economical option.

In this study, we also examined metagenomic sequencing for its ability to detect a genetically modified pathogen in a clinical sample. A BSL-2 strain of *B. anthracis* containing an inserted RFP gene was used as a surrogate for a genetically modified organism (GMO), and spiked into human blood at relatively high concentration. In this case, although reads were identified as likely originating from *B. anthracis*, in none of the samples was evidence of the inserted gene detected. This likely means that, for the time being, even with their substantial output, the benchtop sequencers are not suitable for detection of GMO from complex samples or characterization of threat agents from complex samples. Our results indicate, however, that although *characterization* of a given pathogen from a clinical sample by metagenome sequencing on a benchtop sequencer may not be possible without some pathogen-specific enrichment, *identification* of species and even strains is possible.

The results of this study suggest that the benchtop sequencers perform well at the task of identifying a putative pathogen present at low titers. Each of the three platforms tested provided a number of reads that was sufficient to unambiguously identify the pathogen to species level in the case of virus and to the genus level in the case of bacteria. The data from each platform was very reproducible for technical replicates within library preparations. Indeed, the data from each platform was remarkably consistent in terms of quantity and quality (Table 2; Additional file 2: Table S1). Additionally, there was little variation in the number of reads mapped or in taxonomic classification of contigs. These results suggest that library protocols and sequencing chemistries are robust and uniform enough to make a dependable identification of a given pathogen. Our results are in agreement with a recently published study in which gDNA from *Bacillus anthracis* was serially titrated into background DNA collected from air filters and soil. The results of this study demonstrated that, even with whole genome amplification prior to sequencing, it is difficult to assign a proper species classification to sequence reads from *B. anthracis *[25].

We did note several platform-specific variations in our data. For instance, in the case of the Influenza data from the PGM platform, segment 5 routinely exhibited coverage bias as compared to larger segments. There could be several possible explanations for this observation. The G/C-content of the Influenza A genome displays wide intra-segment and inter-segment variations (Additional file 4: Figure S2). A closer examination of the mapped reads seems to show a slight correlation with areas of low G/C-content, but follow on experiments would be required to conclusively elucidate the impact of G/C-content in this context. A number of studies have noted that NGS data have distinct biases in areas of high G/C-content [17,26,27]. Additionally, template amplification via emulsion PCR is a potential source of reduced library diversity [28]. Moreover, inefficiencies during reverse transcription due to RNA secondary structure may be responsible for the observed coverage bias. Given that the initial step in PGM library construction involves random fragmentation with RNase III at 37°C, it is possible that some RNA strands did not completely unfold. This may be especially true for those segments with higher ΔG, such as segments 1, 2 and 5 (Additional file 4: Figure S2). Although the MiSeq replicates also showed a slight skew towards segment 5, the preference was not as extreme as that for the PGM. This may be due in part to the method for RNA library construction on the MiSeq platform. The initial step involves a chemical fragmentation step at high temperature. Thus, the diversity of the libraries may be different from the start. On the other hand, the PGM replicates demonstrated some variation in mapped reads (Figure 3) suggesting that increased throughput might produce greater diversity.

Another platform-specific characteristic observed in this study was the proportion of mismatches within a non-perfectly mapped read. This statistic was slightly higher for the MiSeq platform than for the PGM platform. Ideally, given the correct reference sequence, for a given platform this statistic would approach zero as sequencing error decreased. However, as in this case, this statistic may also be affected by nucleotide differences between the sequenced strain and the closest reference sequence available from NCBI, precluding us from making any conclusions as to sequencing error rates from these data. It is possible that, to some extent, the longer length of MiSeq reads allows for more opportunity for mapping of non-perfect matches, and this may contribute to a decreased LoD for MiSeq when mapping to a closely related but not identical reference sequence. However, the extent to which error rate versus strain level differences and read length affect this statistic as well as the LoD cannot be ascertained in the absence of a true reference sequence for the strain in question.

A number of recent reports have attempted to define the limitations of metagenomic sequencing data. One study made use of simulated data sets to compare assemblies from three sequencing technologies (Sanger, pyrosequencing and Illumina). Unsurprisingly, the study concluded that assembly quality decreased rapidly with increasing sample complexity. For low complexity samples (10 genomes) the assemblies were comparable in quality and inclusiveness, while Illumina data produced superior assemblies in a higher complexity sample (100 genomes) [29]. These results mirror those presented here in that no one sequencing chemistry clearly surpassed another in terms of identification of a microorganism present in a low-complexity sample. It should be noted that the Illumina data in the study by Mende et al. were from the HiSeq platform and were extensively trimmed to provide high quality reads as input [28]. A separate report estimated that genome coverage of 20X was required for proper taxonomic classification of species present in a given metagenomic community [30]. This study is in agreement with our inability to make a correct species-level determination from our *Bacillus anthracis* samples. Additionally, our results complement an important conclusion from the previous report - that the efficiency of gene detection is most likely overestimated.

There has been much effort to understand and improve metagenomic data from complex samples comprised mostly of bacterial species. There are fewer published studies examining the effects of different sequence technologies on viral metagenomics. A recent research paper attempted a comparison of Roche-454 and Illumina data for estimation of diversity in viral quasispecies, in this case HIV. The authors of that study noted that the increased throughout and lower error rate of the Illumina platform enabled improved reconstruction of viral haplotypes. However, due to the longer read lengths, the Roche-454 was superior when long range reconstruction was necessary [31].

Our results indicate that library diversity and overall throughput are the two key metrics in determining how a researcher or clinician may use metagenomic data from benchtop sequencers. For instance, a recent survey of Human papillomavirus DNA present in human skin tumors demonstrated that the increased throughput of the PGM enabled identification of seven additional viral subtypes as compared to data from Roche-454 sequencing of the same samples [32]. This result is similar to our observation that PGM data provided steady, reproducible identification of Influenza A virus in comparison to sequence data produced by the Roche-454. Overall, the MiSeq proved superior to both the Ion Torrent PGM and Roche-454 for both detection as well as classification of the pathogen present in our mock samples.

Although there is no published report of this, it remains formally possible to identify a previously unknown agent from a single novel microbial read present in a complex metagenomic sample. Indeed, identification of novel agents has been reported with as few as 14 reads out of over 100,000 [33]. Whereas *identification* of an agent may require detection of only one or more reads, *characterization*, the crucial next step, is absolutely dependent on complete (100%) or nearly complete representation of the agent’s entire genome at adequate depth of coverage, especially in the case of RNA viruses or other microorganisms likely to exhibit functionally relevant minority populations or quasispecies, or genetically modified organisms. In this case, it is necessary for follow-on experiments to more fully characterize the genome of the microorganism, such as Sanger sequencing using primers based on the novel fragment(s). It would be optimal if some of the original sample were available for such experiments. However, in many cases, the original sample may be precious or limited in terms of volume. This challenge can be more pronounced when identifying viral agents as opposed to bacterial agents. Viral genomes are orders of magnitude smaller (~1X10^4^-1X10^5^ bps) than those of an average bacterial agent (~3-5X10^6^ bp). Thus, the overall amount of viral nucleic acid may be in the picogram range, increasing the likelihood of two technical obstacles: 1) viral nucleic acid is outcompeted during amplification by other nucleic acids in the matrix, such as host ribosomal RNA if the matrix is tissue or, 2) if the overall amount of nucleic acids in the metagenome sample itself is low, then library preparation of the sample may fail as successive losses of genetic material occur in each step. Targeted amplification of organism-specific regions prior to sequencing has shown some promise. For instance, an assay in which multiplex PCR preceded sequencing was able to fully differentiate *Bacillus anthracis, Yersinia pestis* and *Francisella tularensis*[34].

## Conclusions

In this study, we sought to determine empirical limits of detection for metagenomic sequencing of clinical samples using three different sequencing platforms. We found that the analytical sensitivity of all three platforms approaches that of standard qPCR assays. Although all platforms were able to detect pathogens at the levels tested, there were several noteworthy differences. The Roche-454 Titanium platform produced consistently longer reads, even when compared with the latest chemistry updates for the PGM platform. The MiSeq platform produced consistently greater depth and breadth of coverage, while the Ion Torrent was unequaled for speed of sequencing. None of the platforms were able to verify a single nucleotide polymorphism responsible for antiviral resistance in an Influenza A strain isolated from the 2009 H1N1 pandemic. Additionally none of the platforms tested was able to detect evidence of genetic engineering in a bacterial biowarfare agent that was spiked into a clinical-type sample. Overall, the benchtop platforms perform well for identification of pathogens from a representative clinical sample. However, our results indicate that, unlike identification, *characterization* of pathogens is likely to require higher titers, multiples libraries and/or multiple sequencing runs.

## Methods

### Cells and viruses

*Bacillus anthracis* [34F2 (NCBI taxon ID #526966)] used in this study was routinely cultured in Brain Heart Infusion (BHI) broth (KD Medical; Columbia, MD). Cell counts were performed using the track dilution method [35] on BHI agar plates. One mL aliquots of plaque-purified Dengue virus Type 1 and Type 2 were maintained at -80° until spiking blood. Influenza A virus stocks were purchased from ATCC (Manassas, VA): VR-1683(NCBI taxon ID # 380342) and VR-1736 (NCBI taxon ID # 710659).

### Sample preparation

#### *(i) Viral samples*

Frozen aliquots of plaque-purified Dengue virus Type 1, Dengue virus Type 2, or Swine Influenza A were ten-fold serially diluted in sterile, 1X PBS. One mL aliquots of sodium citrate-treated whole human blood (BioReclamation, Liverpool, NY) were spiked with 100 μL of diluted virus at various titers. After thorough mixing with a micropipette, Trizol LS (Life Technologies, Grand Island, NY) was added to the sample at a ratio of 3:1. Samples were processed for total RNA according to manufacturers’ protocol.

#### *(ii) Bacterial samples*

Log-phase cultures of *B. anthracis* strain 34F2^RFP were ten-fold serially diluted in 1X sterile PBS. Two mL of sodium citrate-treated whole blood were spiked with 200 μL of diluted culture at various titers. After thorough mixing with a micropipette, genomic DNA (gDNA) was extracted with a BioStic® Bacteremia DNA Isolation kit (Mobio; Carlsbad, CA) according to manufacturers’ protocol.

### Nucleic acid quality checks

RNA integrity and purity were assayed using an RNA 6000 Pico chip on the Agilent Bioanalyzer™. RNA mass was determined by fluorescent detection using Qubit® Broad Range RNA kit (Life Technologies). All RNA samples were stored at -80°C until use. DNA integrity and purity were assessed by agarose gel electrophoresis on 0.8% E-Gels® (Life Technologies). DNA mass was determined using Qubit® Broad Range DNA kit (Life Technologies). All DNA samples were stored at -20°C until use.

### High-throughput sequencing

#### *(i) Roche-454 pyrosequencing*

For RNA samples, libraries were constructed by following the cDNA Rapid protocol (Roche Diagnostics, Mannheim Germany) using an input of 200 ng of total RNA. For DNA samples, libraries were constructed following the Rapid DNA protocol (Roche) using an input of 500 ng of gDNA. All emPCR reactions were performed using GS FLX Titanium Lib-L-LV kits. Template-to-bead ratios were optimized via titration. All sequencing runs were performed using a two-region gasket for each pico-titre plate. Each run was 200 cycles.

#### *(ii) Ion Torrent PGM® sequencing*

For RNA samples, libraries were constructed by following the Whole Transcriptome Library protocol using an input of 500 ng total RNA. For DNA samples, libraries were constructed using the IonXpress™ Plus gDNA Fragment Library protocol using an input of 500 ng gDNA. Quality control of all libraries was performed on the Agilent Bioanalyzer using a High Sensitivity chip. Library templates were clonally amplified using the Ion One Touch 2™, following the manufacturers’ protocol. Template dilutions were calculated by extrapolation from a qPCR standard curve using the Ion Library Quantitation Kit (Life Technologies). Recovered template-positive Ion sphere particles (ISPs) were subjected to enrichment according to template corresponding protocol. Ion Sphere quality control was performed on enriched and un-enriched template ISPs using the Ion Sphere™ Quality Control Kit (Life Technologies). Samples containing an optimum number of template ISPs and satisfactory enrichment were subjected to the standard Ion PGM™ 200 Sequencing v2 protocol.

#### *(iii) Illumina MiSeq sequencing*

Illumina TruSeq cDNA libraries were prepared from 400 ng total RNA, omitting the polyA selection step. Each library was subjected to a full MiSeq run using a 300 cycle kit, paired end sequencing. A quality control tool for high throughput sequence, FASTQC, a java stand-alone program, was downloaded from Babraham Bioinformatics Institute: http://www.bioinformatics.babraham.ac.uk/projects/fastqc/ and each fastq file was checked for quality.

### Quantitative PCR

#### *(i) RNA samples*

Detection and quantification of Dengue virus and Influenza A virus in samples was performed with SYBR® Green-based real time RT-PCR. Equal masses of total RNA from each sample were analyzed in duplicate using Express One SYBR® GreenER™ (Life Technologies) following manufacturer’s instructions. Specificity of primer pairs (Additional file 5: Table S3) for each virus was checked using NCBI Primer BLAST against the nr database. Standard curves for Dengue virus were constructed using ten-fold serial dilutions of viral RNA extracted from a stock aliquot and are expressed in pfu/mL. Standard curves for Influenza A virus were constructed using a 250 base RNA oligo (Bio-Synthesis Inc., Lewisville, TX) representing the region of the gene to be amplified. Influenza A values are expressed in genome copies/mL.

#### *(ii) DNA samples*

Detection and quantification of *Bacillus anthracis* in samples was performed using SYBR® Green-based real time PCR. Equal masses of gDNA were analyzed in duplicate using Power SYBR® Green (Applied Biosystems, Grand Island, NY). Specificity of primer pairs (Additional file 5: Table S3) for each gene was checked using NCBI Primer BLAST against the nr database. Standard curves for each gene were constructed using ten-fold serial dilutions of a plasmid construct containing the complete coding sequence of each gene. Values are expressed in copies/mL.

### Bioinformatics

#### *(i) Reference mapping*

Sequence data mapping was performed using CLC Genomics Workbench v6.0.4 (CLC Inc, Aarhus, Denmark). CLC Reference Mapper was run with default settings (Insertion cost = 3, Deletion cost = 3, Mismatch cost = 2, Length fraction = 0.5 and similarity fraction = 0.8). CLC default settings were arbitrarily assigned as low stringency settings. Medium stringency settings were arbitrarily defined as Insertion cost = 3, Deletion cost = 3, Mismatch cost = 2, Length fraction = 0.7 and similarity fraction = 0.8. High stringency settings were defined as Insertion cost = 3, Deletion cost = 3, Mismatch cost = 3, Length fraction = 1.0 and similarity fraction = 1.0. GS Reference Mapper software (v2.0.01.14; Roche/454) was used to produce reference-guided assemblies of each of the Roche datasets with respect to the DENV-1 genome (GenBank; DVU88536).

#### *(ii) de novo assembly and BLASTn analysis of contigs*

MIRA V3.4.0.1 production version was used for the assembly of PGM data. The SFF file was converted to fastq using the sff_extract.py utility. The job parameter was modified with the keywords “denovo,genome,accurate,iontor” to indicate a *de novo* assembly for the corresponding platform. In addition, the modifier “-GE:not” was set to 12 processors for parallel computing. The assembly parameters for MIRA for the PGM data were, minimum overlap = 17 bps, minimum contig size =150 bps, minimum neighbor quality needed for tagging = 20, minimum read length =80. For the Velvet assembly the k-mer size was 33.

The assembly of the MiSeq data was conducted using velvet version 1.2.1. The velvet assembly was conducted using a k-mer of size 31 and “short” modifier. For the downstream analysis, BLASTN and annotation, only those contigs with size greater than 150 bps were considered.

BLASTN was conducted with the NCBI-BLAST++ version 2.2.28 against the NT BLAST database from 04/22/2013. The taxonomical annotation was obtained with an in-house script and the results were visualized using MEGAN version 4.70.4.

#### *(iii) Mathematical modeling*

Given a particular genome (G) of size (N), we were interested in a particular gene (H) of size (M) within G. If a sequencing experiment is conducted and a particular read from the experiment matches the sequence of H, this is labeled a hit. In order to examine the range of read counts that will result in a range from one hit with low probability to one hit with near certainty, we decided to recreate the parameters of a sequencing experiment *in silico*. The simulation was written in PERL, where N was set to 5.23 Mbps, M was set to 1000 bps, and five copies of H were used in the simulation. The simulation assumes that all parts of G are equally likely to be sequenced. A thousand iterations of the simulation produced the expected number of hits for read counts of 1 k, 2 k, 3 k, 4 k, 5 k, 8 k, 10 k and 15 k.

## Ethics

No research regarding human or animal subjects was conducted at NMRC or USUHS as part of this work. Human blood was obtained commercially as a laboratory reagent (and in the absence of all identifiers) from BioreclamationIVT (http://www.bioreclamation.com) and therefore did not require institutional review by an ethics committee at NMRC. Bioreclamation collects blood from healthy, consented, paid human donors.

## Competing interests

The authors declare no competing financial or non-financial interests.

## Authors’ contributions

KGF and KABL conceived of and designed the study. KGF and SLS prepared the samples. KGF, CLR and TL performed the sequencing. KGF, JEHG and KABL analyzed the data. JEHG performed the *de novo* assemblies and taxonomic classification of the contigs. KGF and KABL wrote the manuscript with input and edits from VM, AJM and JEHG. All authors read and approved the final manuscript.

## Supplementary Material

Additional file 1: Figure S1 Read mapping against segment 5 of Influenza genome. Reads resulting from Ion Torrent 314 chip were mapped to the reference Influenza a H1NI segment 5 [NCBI accession: NC_002019], using CLC Genomics Workbench version 6.0 at default parameters. Coordinates of reference genome segment are displayed along the top and G/C content is graphed below reference in pink. Click here for file

Additional file 2: Table S1 Mapped reads by Influenza A segment for MiSeq and PGM replicates^a^. a: Statistics for one of two independent libraries at low stringency parameters. Click here for file

Additional file 3: Table S2 Proportion of mapped reads as a function of Influenza A genome segment size for MiSeq and PGM replicates^a^. a: Statistics for one of two independent libraries at low stringency parameters. Click here for file

Additional file 4: Figure S2 Predicted free energy of individual Influenza genome segments. Using CLC Genomics Workbench version 6.0, Gibb's free energy was predicted for each genome segment of the NCBI reference strain Influenza A virus (A/Puerto Rico/8/34(H1N1)). Click here for file

Additional file 5: Table S3 Sequences of primers used in this study. Click here for file
