# Supplementary figures and images for: Comparison of three next-generation sequencing platforms for metagenomic sequencing and identification of pathogens in blood
Source: BMC Genomics. 2014 Feb 4;15:96. doi: 10.1186/1471-2164-15-96 (PMC3922542; doi:10.1186/1471-2164-15-96)

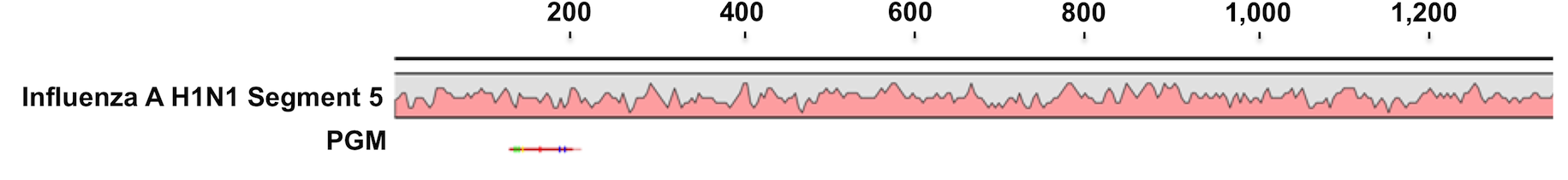

Supplement: Additional file 1: Figure S1 — Read mapping against segment 5 of Influenza genome. Reads resulting from Ion Torrent 314 chip were mapped to the reference Influenza a H1NI segment 5 [NCBI accession: NC_002019], using CLC Genomics Workbench version 6.0 at default parameters. Coordinates of reference genome segment are displayed along the top and G/C content is graphed below reference in pink. [file 1471-2164-15-96-S1.png]

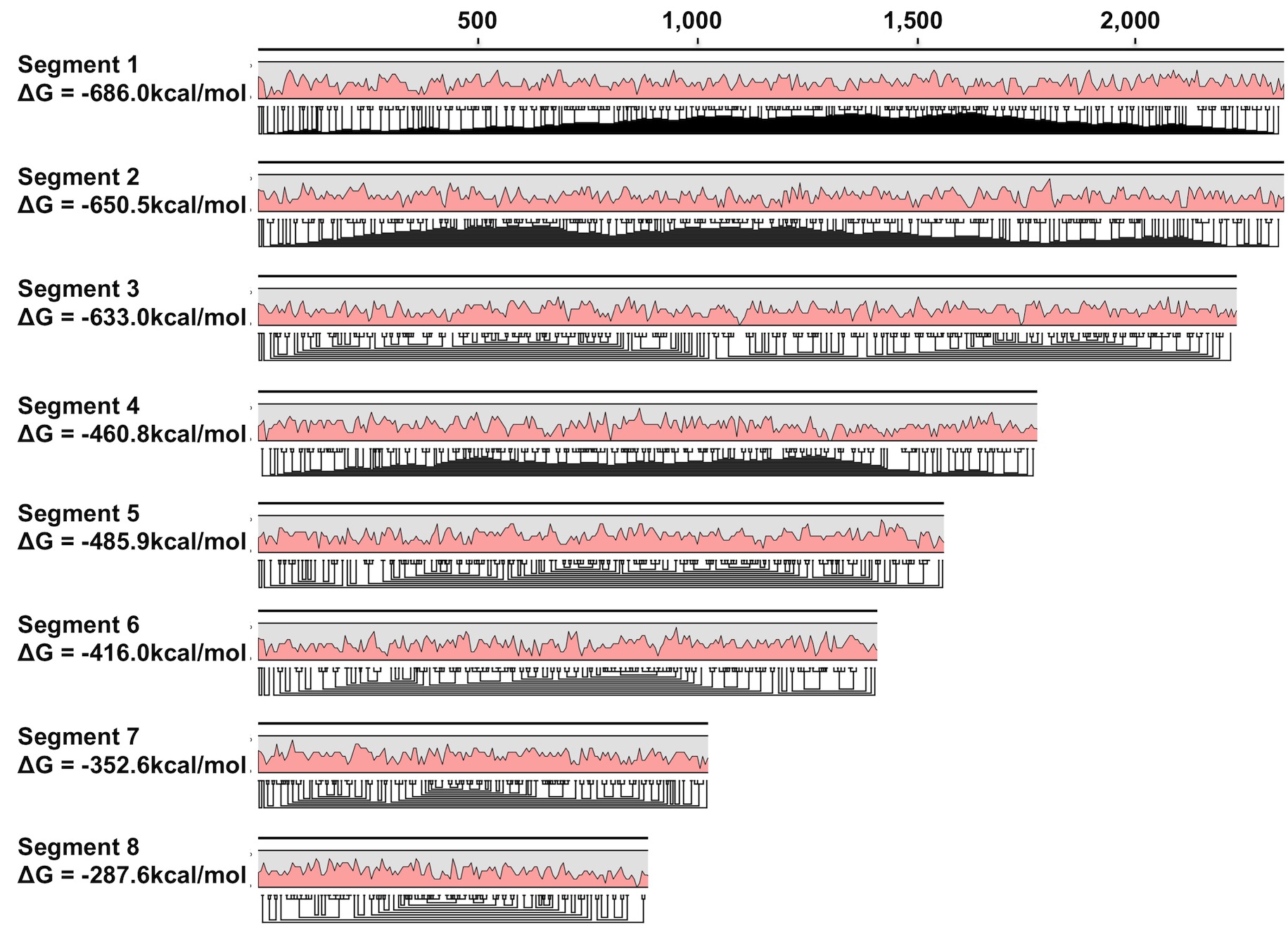

Supplement: Additional file 4: Figure S2 — Predicted free energy of individual Influenza genome segments. Using CLC Genomics Workbench version 6.0, Gibb's free energy was predicted for each genome segment of the NCBI reference strain Influenza A virus (A/Puerto Rico/8/34(H1N1)). [file 1471-2164-15-96-S4.png]
