# Supplementary material for: Comparison of three next-generation sequencing platforms for metagenomic sequencing and identification of pathogens in blood
Source: BMC Genomics. 2014 Feb 4;15:96. doi: 10.1186/1471-2164-15-96 (PMC3922542; doi:10.1186/1471-2164-15-96)
Supplement: Additional file 2: Table S1 — Mapped reads by Influenza A segment for MiSeq and PGM replicatesa. a: Statistics for one of two independent libraries at low stringency parameters. [file 1471-2164-15-96-S2.docx]

| **Segment (Gene)** | **MiSeq 1** | **MiSeq 2** | **MiSeq 3** | **PGM 1** | **PGM 2** | **PGM 3** |
| --- | --- | --- | --- | --- | --- | --- |
| 1 (PB2) | 44 | 58 | 53 | 2 | 2 | 2 |
| 2 (PB1) | 60 | 55 | 68 | 2 | 4 | 4 |
| 3 (PA) | 40 | 30 | 33 | 2 | 0 | 2 |
| 4 (HA) | 35 | 40 | 45 | 3 | 3 | 1 |
| 5 (NP) | 51 | 40 | 44 | 8 | 5 | 6 |
| 6 (NA) | 29 | 39 | 32 | 1 | 2 | 0 |
| 7 (M1/M2) | 31 | 38 | 33 | 2 | 1 | 3 |
| 8 (NS1/NS2) | 18 | 22 | 15 | 1 | 1 | 1 |

a: Statistics for one of two independent libraries at low stringency parameters
