# Supplementary material for: Comparison of three next-generation sequencing platforms for metagenomic sequencing and identification of pathogens in blood
Source: BMC Genomics. 2014 Feb 4;15:96. doi: 10.1186/1471-2164-15-96 (PMC3922542; doi:10.1186/1471-2164-15-96)
Supplement: Additional file 3: Table S2 — Proportion of mapped reads as a function of Influenza A genome segment size for MiSeq and PGM replicatesa. a: Statistics for one of two independent libraries at low stringency parameters. [file 1471-2164-15-96-S3.docx]

**Additional File 3 –Proportion of mapped reads as a function of Influenza A genome segment size for MiSeq and PGM replicates^a^**

| **Segment (Gene)** | **MiSeq 1** | **MiSeq 2** | **MiSeq 3** | **MiSeq Average** | **PGM 1** | **PGM 2** | **PGM 3** | **PGM Average** |
| --- | --- | --- | --- | --- | --- | --- | --- | --- |
| 1 (PB2) | .06 | .08 | .07 | .07 | .04 | .05 | .04 | .04 |
| 2 (PB1) | .08 | .07 | .09 | .08 | .04 | .09 | .09 | .07 |
| 3 (PA) | .06 | .04 | .05 | .05 | .04 | .00 | .05 | .03 |
| 4 (HA) | .06 | .07 | .08 | .07 | .08 | .09 | .03 | .07 |
| 5 (NP) | .11 | .08 | .09 | .09 | .24 | .18 | .20 | .21 |
| 6 (NA) | .07 | .09 | .07 | .08 | .03 | .08 | .00 | .04 |
| 7 (M1/M2) | .10 | .11 | .10 | .10 | .09 | .05 | .15 | .10 |
| 8 (NS1/NS2) | .07 | .08 | .05 | .07 | .05 | .06 | .06 | .06 |

a: Statistics for one of two independent libraries at low stringency parameters
