# Supplementary material for: Comparison of three next-generation sequencing platforms for metagenomic sequencing and identification of pathogens in blood
Source: BMC Genomics. 2014 Feb 4;15:96. doi: 10.1186/1471-2164-15-96 (PMC3922542; doi:10.1186/1471-2164-15-96)
Supplement: Additional file 5: Table S3 — Sequences of primers used in this study. [file 1471-2164-15-96-S5.docx]

| **Primer** | **Sequence (5’🡪3’)** | **GenBank ref** | **Reference** |
| --- | --- | --- | --- |
| DENV-1 NS5 F | GAC ACC ACA CCC TTT GGA CAA | M87512 | [[23](#_ENREF_23)] |
| DENV-1 NS5 R | CAC CTG GCT GTC ACC TCC AT | “ | [[23](#_ENREF_23)] |
| DENV-2 C F | CAT GGC CCT KGT GGC G | M29095 | [[23](#_ENREF_23)] |
| DENV-2 C R | CCC CAT CTY TTC AGT ATC CCT G | “ | [[23](#_ENREF_23)] |
| InfA PA F | CAG AGC CAC AGA ATA CAT AAT GAA G | M26076.1 | This study |
| InfA PA R | TTG GTC TTT CGC CTT CCC TC | “ | This study |
| InfA HA F | CCA GTC ACA ATA GGA GAG TG | EU139823.1 | This study |
| InfA HA R | AAA CCG GCA ATG GCT CCA AA | “ | This study |
| Ban *sap* F | CAA TCG AAA TGG CTG ACC AAA | NC_005945.1 | This study |
| Ban *sap* R | ACC CTC TGG TGA AAC AAC TTC AGT | “ | This study |
| RFP F | TCG CTG GTT TCT TTG AGC GAT ACG | EF606900.1 | This study |
| RFP R | CAC GTG GCT GGG AGG CGA AT | “ | This study |
